# Scaling-up strategic purchasing: analysis of health system governance imperatives for strategic purchasing in a free maternal and child healthcare programme in Enugu State, Nigeria

**DOI:** 10.1186/s12913-018-3078-x

**Published:** 2018-04-05

**Authors:** Daniel Chukwuemeka Ogbuabor, Obinna Emmanuel Onwujekwe

**Affiliations:** 1 Department of Health Systems and Policy, Sustainable Impact Resource Agency, 22 Ogidi Street, Asata, P.O. Box 15534, University of Nigeria Enugu Campus (UNEC), Enugu, Enugu State Nigeria; 2 Department of Health Administration and Management, University of Nigeria Enugu Campus, Enugu, Enugu State Nigeria; 3 Health Policy Research Group, College of Medicine, University of Nigeria Enugu Campus, Enugu, Enugu State Nigeria

**Keywords:** Governance, strategic purchasing, Free healthcare, Policy implementation, Nigeria

## Abstract

**Background:**

Significant knowledge gaps exist in the functioning of institutional designs and organisational practices in purchasing within free healthcare schemes in low resource countries. The study provides evidence of the governance requirements to scale up strategic purchasing in free healthcare policies in Nigeria and other low-resource settings facing similar approaches.

**Methods:**

The study was conducted at the Ministry of Health and in two health districts in Enugu State, Nigeria, using a qualitative case study design. Semi-structured interviews were conducted with 44 key health system actors (16 policymakers, 16 providers and 12 health facility committee leaders) purposively selected from the Ministry of Health and the two health districts. Data collection and analysis were guided by Siddiqi and colleagues’ health system governance framework. Data were analysed using a framework approach.

**Results:**

The key findings show that supportive governance practices in purchasing included systems to verify questionable provider claims, pay providers directly for services, compel providers to procure drugs centrally and track transfer of funds to providers. However, strategic vision was undermined by institutional conflicts, absence of purchaser-provider split and lack of selective contracting of providers. Benefit design was not based on stakeholder involvement. Rule of law was limited by delays in provider payment. Benefits and obligations to users were not transparent. The criteria and procedure for resource allocation were unclear. Some target beneficiaries seemed excluded from the scheme. Effectiveness and efficiency was constrained by poor adherence to purchasing rules. Accountability of purchasers and providers to users was weak. Intelligence and information is constrained by paper-based system. Rationing of free services by providers and users’ non-adherence to primary gate-keeping role hindered ethics.

**Conclusion:**

Weak governance of purchasing function limits potential of FMCHP to contribute towards universal health coverage. Appropriate governance model for strengthening strategic purchasing in the FMCHP and possibly free healthcare interventions in other low-resource countries must pay attention to the creation of an autonomous purchasing agency, clear framework for selective contracting, stakeholder involvement, transparent benefit design, need-based resource allocation, efficient provider payment methods, stronger roles for citizens, enforcement of gatekeeping rules and use of data for decision-making.

**Electronic supplementary material:**

The online version of this article (10.1186/s12913-018-3078-x) contains supplementary material, which is available to authorized users.

## Background

Enugu State, Nigeria, introduced tax-funded free maternal and child healthcare programme (FMCHP) in December 2007 to improve use of primary health care, ensure that households are protected against the financial risk of obtaining essential maternal and child healthcare and reduce maternal and child mortality [1]. A striking feature of the FMCHP is a mutually co-existing two-tier public health system - free maternal and child healthcare services are obtained exclusively from publicly owned health facilities in a system that usually charges user fees. Service entitlement was automatic initially, but since 2011, has been linked to presentation of evidence of tax payment. When users do not present evidence of taxation, they pay user fees. Evidence of tax payment was introduced as a rationing strategy to limit the use of free services to residents of Enugu state but is not a convention for securing fee-paying services.

**Fig. 1:**
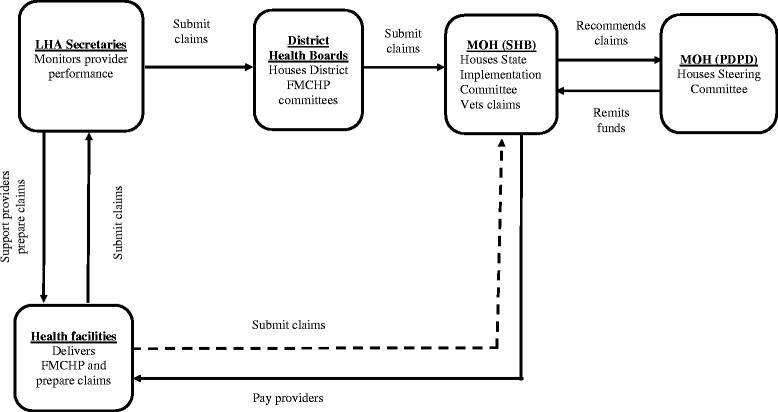
Institutional design for purchasing in FMCHP in Enugu State. Legend:  Teaching hospital

The FMCHP is governed through the district health system in which the state is delineated into 7 districts and 68 Local Health Authorities and the Ministry of Health is structured into a policy arm, the Policy Development and Planning Directorate (PDPD), and a service delivery agency, the State Health Board (SHB). A multi-stakeholder governing body, the Steering Committee, housed within the PDPD manages the FMCHP, pools FMCHP fund and serves as the primary purchaser. The State Implementation Committee, housed within the SHB, acts as the secondary purchaser. An output-based purchasing approach is adopted, where the FMCHP committees remunerated health facilities per patient who received free services as shown in Fig. 1. The State Implementation Committee receives and verifies provider claims, which consist of drug costs and service charges, within 4 weeks guided by the approved fee schedule. Claims that follow the purchasing rules are recommended to the Steering Committee for approval. The Steering Committee remits approved claims to the State Implementation Committee, who should pay providers within 1 week of receipt of funds. While 70% of service claims are paid to health facilities, 30% are allocated to the district health system structures to cover their administrative costs. The approved drug claims are credited to health facilities at the central medical stores.

Good health system governance is essential to optimal implementation of policies to scale up strategic purchasing for universal health coverage (UHC) schemes [2]. A purchasing system consists of benefit package design, provider payment systems and institutional structures underpinning the provider payment systems [3]. Benefit package design refers to which healthcare goods, services and intervention to buy, service exclusions and cost-sharing by covered population at the point of use [4]. Assigning purchasing function to an institution, termed the purchasing agency, and the set of rules regulating how that institution relates to other health system actors constitute the institutional structures; while the provider payment systems are methods for transferring funds from a purchaser of health services to the providers [3, 4]. Passive purchasing, which is based on historical norms, differs from strategic purchasing defined as the transfer of revenue to selected providers based on information on population health needs, active stakeholder engagement and provider performance [5]. Governance for strategic purchasing involves setting the general rules, which define who sets specific purchasing rules, how and when these rules can be changed, provision of strategic direction and coordination for different health financing actors [6]. Governing strategic purchasing promotes efficiency, quality and equity by enhancing interactions among actors and elements that characterize the purchasing system [2, 4, 5, 7].

Whereas purchasing in free healthcare policies applies to a specific component of the health system and has been relatively unexplored, existing scholarship has focused on purchasing in tax-financed UHC schemes which apply to a whole system. Purchaser-provider split, described as assigning the purchasing and provision of healthcare services to separate organisations [3], was achieved in user fee removal schemes in Thailand, India, Vietnam and Nigeria [8–12], but undermined in Mexico, where the State Ministries of Health usurped purchasing function assigned to the State Health Social Protection Regime (REPSS) [13]. In China’s New Cooperative Rural Medical Scheme (NCMS), local government had autonomy to implement the scheme, determine the level of subsidy, the extent of benefit package and the co-payment stewardship, whereas the stewardship and fund management were assigned to the coordinating team and fund management committee respectively [14].

Evidences on governing benefit package design and selective contracting of providers, defined as choosing providers that meet the minimum accreditation requirements [3], are mixed. Stakeholder participation and transparency in the benefit package design in tax-funded fee exemption schemes were high in Thailand and Mexico, but low in Vietnam, India, Nigeria and China [10–12, 14, 15]. Health technology assessment (HTA) informed the benefit design in Thailand and Mexico, but not in India and Nigeria [11, 12, 15, 16]. Provider accreditation, has been used to contract providers, monitor and improve quality of care in tax-funded fee exemption schemes in Thailand and China [4, 8, 14, 15], but is limited in scope and transparency in Mexico’s Seguro Popular [9] and totally absent in a free healthcare policy in Nigeria [12].

Provider payment methods have not been consistent across user fee removal schemes in different countries. Vietnam adopted group specific capitation with a cap on provider payment and from which referrals costs are paid using fee-for-service [11]. In Thailand’s universal coverage scheme (UCS), age-adjusted capitation is used for outpatient and diagnostic related groups (DRG) with fixed annual budget and cap for inpatients [8, 17]. Ghana and Mexico adopted fee-for-service, but whereas fee-for-service in Mexico’s Seguro Popular remained unchanged, Ghana’s user fee exemption scheme transited from fee-for service to diagnostic related groups (DRG) method [9, 18]. In Nigeria, paying primary care providers using global capitation and referrals through fee-for-service provided incentive for purchasers to delay referrals from primary to secondary facilities [12].

Significant knowledge gaps exist in the functioning of institutional designs and organisational practices in purchasing within free healthcare schemes in low resource countries. The Mandates of the different actors in the FMCHP purchasing system in Enugu state seem unclear. Even though institutional conflicts seem to affect the benefit design, provider payment and institutional structures for purchasing, little is known of the key governance requirements for moving to more strategic purchasing. The purpose of this study, therefore, is to identify, from Enugu’s experiences, the governance requirements to scale up of strategic purchasing in free healthcare policies in low-resource settings facing similar approaches.

## Methods

### Conceptual framework

The study was guided by the Siddiqi and colleagues health systems governance framework [19]. The framework uses ten governance principles - strategic vision, participation and consensus orientation, rule of law, transparency, responsiveness, equity and inclusiveness, effectiveness and efficiency, accountability, information and intelligence, and ethics – to explore institutional designs and organisational practices within health systems. We adapted these fundamentals of health systems governance to explore purchasing in the free healthcare policy in Enugu State, Nigeria. This framework was chosen because it provides useful analytical tool for investigating the enablers and constraints to health systems governance at policy and operational levels and could inform interventions that improve the performance of health systems [19].

### Study setting

The study was conducted at the Ministry of Health and in two districts (District A = Isi-Uzo and District B = Enugu Metropolis) in Enugu State, South-east Nigeria. As in most of Nigeria, conditions that predict financial catastrophe including healthcare cost paid out of pocket, households’ inability to pay and absence of prepayment mechanisms to pool financial risks are prevalent in Enugu State [20, 21]. Table 1 shows the selected characteristics of the case study districts [22–26].

**Table 1:** Important characteristics of case study districts

Characteristics	District A	District A
Geographical location	Rural	Urban
Total Population (2016 projection)^a^	203,364	990,225
Growth rate^a^	3.2%	3.2%
Population of women of child bearing age (WCBA) (46.9% urban; 42.8% rural)^b^	87,040	464,416
Population of U-5 children (15.5% urban; 18.1% rural)^b^	36,809	153,485
% of WBCA currently pregnant (9.5 in urban, 14% in rural)^b^	12,186	44,120
Proportion delivered in public health facility (36.5%)^b^	4448	16,104
Number of public PHC facilities^c^	31	46
Number of public SHC facilities^c^	3	6
Number of private health facilities^c^	6	168
Number of Health facility committees^d^	20	8
Mean nurses per PHC facility^e^	0.09	0.44
Mean number of CHEWs/ JCHEWs per PHC^e^	3.3	10.9

Sources: ^a^Based on Nigeria 2006 Census report [22], ^b^NDHS (2013) [23], ^c^Enugu State Referral Directory [25], ^d^Health Facility Committee Report [24] and ^e^Nkwo et al. [26]

### Research design

The study adopted a qualitative, case study design because experiences of implementation are embedded in the contextual factors that form the focus of this study [27].

### Study population and sampling strategy

State-level policymakers and district-level actors, whose posts included FMCHP implementation, were involved in this study. The state-level policymakers (*n* = 12) were purposively selected from the Ministry of Health based on availability and willingness to participate in the study. The seven health districts were divided into two clusters of well-performing and poor-performing districts based on reimbursement of providers [28]. One district was selected from each cluster by simple random sampling. The district-level policymakers (*n* = 4), providers (*n* = 16) and health facility committee leaders (*n* = 12) were purposively selected. We purposively selected the two busiest public hospitals and six primary health centres that have active health facility committees in each district.

### Data collection

We interviewed 44 participants between February and September 2015 using pre-tested, in-depth, semi-structured interview guide (for details see Additional file 1) as a part of large assessment of governance of the FMCHP [28]. The interview questions were informed by the Siddiqi and colleagues’ governance framework [19], adapted to purchasing concerns in the FMCHP (Table 2). The participants were identified using government officials and health facility staff as gatekeepers. The interviews, which held in offices or health facilities, were conducted in English and lasted at most 90 min. All interviews were audiotaped and transcribed verbatim. The transcripts were sent back to participants to verify accuracy of transcription [29].

**Table 2:** Applying Siddiqi et al. [19] governance framework principles to purchasing in FMCHP

Principle	Domains
Strategic vision	
Strategic vision means that actors should have strategic direction with clear priorities, roles and performance targets; and a shared long-term goal and strategic plan	Organisational autonomy of purchasing agency and providers. Selective contracting with providers
Participation and consensus orientation	
People should have voice in decision-making for health, either directly or through their legitimate intermediate institutions that represent their interest	Participation in implementation of evidence of tax payment. Engagement of Local Health Authority Secretaries in reimbursement process.
Rule of Law	
Legal frameworks pertaining to health and standards, guidelines, policies, and regulations should be fair and consistently enforced.	Enforcement of reimbursement standards
Transparency	
Processes, institutions and information needed to monitor health matters are directly accessible to relevant health system actors when and where they are needed.	Transparency of benefit package design and reimbursement of providers.
Responsiveness	
Institutions and processes should try to serve all stakeholders to ensure that policies and programs are responsive to health and non-health needs of its users	Policy modification through implementation, resource gaps and implications.
Equity and inclusiveness	
All men and women should have opportunities to improve or maintain their health and well-being	Equity in access to free care
Effectiveness and efficiency	
Processes and institutions should produce results that meet population needs and influence health systems outcomes without waste of resources	Organisational capacity of Steering and Implementation Committees of FMCHP
Accountability	
Public officials and service providers are answerable to the public and institutional stakeholders for processes and outcomes.	Citizen-driven accountability in purchasing
Intelligence and information	
Timely generation, collection, analysis and dissemination of accurate information to provide evidence for informed decisions that influence behaviour of different health system actors.	Availability of information technology-driven provider payment system. Generation and use of data for wider system monitoring and decision-making.
Ethics	
Policies and institutional mechanisms should promote and enforce high ethical standards in healthcare and safeguard interests and rights of patients.	Rationing of free services and ethical standards of care.

### Data analysis

Data were analysed using a framework approach [30]. The transcripts were anonymised and imported into NVivo 11 software to facilitate analysis. We used both deductive and inductive coding strategies. The main themes were deductively developed and aligned with ten dimensions of health system governance framework. Inductive codes reflected purchasing functions and institutions; and were generated by familiarization with data and assigning codes to emerging themes (Table 3). The coding of transcripts was carried out by two independent coders and inconsistencies resolved by consensus. The findings were validated in a stakeholders’ meeting.

**Table 3:** Governance practices in FMCHP in Enugu State

Principles	Themes	Sub-themes
Strategic vision	Autonomy of purchaser and providers	Dysfunctional inter-organisational relationships Lack of purchaser-provider split
	Selection of health facilities	Absence of selective contracting/ accreditation of providers
	Financial oversight	Existence of financial monitoring committee
Participation and consensus orientation	Participation in benefit package design	Weak stakeholder participation in formulation and implementation of evidence of tax payment
	Participation in provider monitoring	Lack of clarity about position of Local Health Authority Secretaries
	Participation in reimbursement process	Disengagement of Local Health Authority Secretaries in reimbursement process.
Rule of law	Enforcement of reimbursement standards	Delays in reporting claims, vetting claims and approval and transfer of funds to providers
		Revised reimbursement process to reduce delays
		Quality assurance visit to health facilities by vetting team
Transparency	Transparency of benefit package design	Misinterpretation of evidence of income tax payment by providers and district-level policymakers
	Transparency in reimbursement process	Inflation of claims by providers, district officials and vetting officers
		Resistance to financial monitoring committee from State Health Board officials
Responsiveness	Need-based resource allocation	Service delivery gaps because resources for free care allocated to providers are not need-based
		Effective return of user fees/ informal payments
Equity and inclusiveness	Equity in access to free care	Rural-urban health workforce imbalances favoured urban areas
		Lower use of free care in rural areas than urban areas due to evidence of tax payment and service delivery gaps
Effectiveness and efficiency	Functioning of FMCHP institutional structures.	Dysfunctional Steering and Implementation Committees of FMCHP
		Ministry of Health and State Health Board usurped functions of Steering and Implementation Committees
		Use of FMCHP funds for other purposes
		Non-existent district implementation committees
Accountability	Citizen-driven accountability	Purchaser and providers weakly accountable to users
		Civil society organisations champion delinking of entitlements from evidence of income tax payment
Intelligence and information	Generation and use of data	Transition of state tertiary hospital from primary care provider to referral centre evidence-driven
		Policy change in reimbursement of providers informed by evidence
		Lack of information technology-driven provider payment system.
Ethics	Ethical standards of care	Preference for fee-paying users by providers
		State tertiary hospital refuses referrals from lower facilities
		Rationing of free services even in emergencies

### Ethical consideration

The study was approved by the Health Research Ethics Committee of the University of Nigeria Teaching Hospital Enugu, Nigeria. Informed consent was obtained from all participants for both participation and audio-recording of interviews.

## Results

### Strategic vision

Almost all policymakers and few providers described a two-tier purchasing system, in which, the PDPD is the primary purchaser; and the State Health Board (SHB) that manages service provision, the secondary purchaser. Policymakers explained that the PDPD and SHB usurped the roles of Steering Committee and State Implementation Committee respectively. It was observed that the Steering Committee met only twice in 7 years; first, to ratify policy change in reimbursement procedure and secondly, to approve use of evidence of tax payment policy. Health facility committee (HFC) leaders were not aware of the roles of FMCHP committees in purchasing but explained that fee-exempt MCH services co-existed with a fee-paying public health system.

Most policymakers and providers noted that there is no system for selecting and accrediting health facilities involved in delivery of the FMCHP. The HFC leaders were not aware of how health facilities are selected. Policymakers claimed that the FMCHP is implemented in all publicly owned health facilities across all the Local Government Areas of the state, but some HFC leaders and providers observed that some health facilities were no longer implementing the FMCHP. One provider observed, *“I don’t provide any free maternal health and child health services here, because the centre’s name is not among the centres that are performing free maternal and child healthcare programme”* (Provider 13, District B).

### Participation and consensus orientation

The benefit package design was not participatory. Participants of all categories held the view that the decision to transit from automatic service entitlement to use of evidence of tax payment as rationing strategy did not involve the district stakeholders. A healthcare provider noted that *“There was a memo directing providers that clients coming for free maternal and child health services must provide evidence of tax payment before receiving free care”* (Provider 9, District B). Most HFC leaders argued that if HFCs were consulted when formulating evidence of tax payment policy, they would have suggested alternative strategies for identifying eligible target beneficiaries.

Lack of clarity about the position of primary health care (PHC) coordinators and Local Health Authority (LHA) Secretaries limited their involvement in the FMCHP and remittance of their share of service costs. A policymaker attested: *“there has always been this confusion about who PHC coordinators, HOD Health and LHA secretaries were”* (Policymaker 1, PDPD). A district-level policymaker explained that *“since state-level policymakers stopped involving Local Health Authority Secretaries in the reimbursement process, the Local Health Authority Secretaries became aloof”* (Policymaker 14, District A).

### Rule of law

Most policymakers and providers stated that unclear procedure and delayed reimbursement procedure constrained provider payment. Three types of reimbursement delays – delay in submission of provider claims, delay in vetting and approval of claims, and delay in transferring funds to providers were revealed, which necessitated revision of the reimbursement standards. Even though most HFC leaders stated that they were not involved in the reimbursement procedure, few HFC leaders indicated that providers no longer submit claims due to unpredictable reimbursement.

The delay in reporting claims was attributed to weak provider capacity to fill reimbursement forms, understaffing of health facilities and lack of support from district-level officials. It was explained that at inception, Local Health Authority (LHA) Secretaries supported providers to complete and submit FMCHP claims. However, there was a change from reimbursement through the district-level structures to direct facility reimbursement of FMCHP service expenditure and crediting of providers’ drug account at the central medical store for FMCHP expenditure on drugs. Consequently, the district officials withdrew facilitation of claims’ preparation and submission after direct facility reimbursement was introduced. As one provider observed:

*“HODs are not usually available and they are required to sign the invoice. The district chiefs are also required to endorse the claims. These are the protocols that must all be observed before you submit the invoice to Enugu…and the process takes such a long time*” (Provider 1, District A).

Policymakers stated that vetting of FMCHP expenditure claims was done as an ad hoc exercise and had no budget line to defray administrative costs nor provide incentives to the vetting team. Members of the vetting team were frequently transferred. Policymakers and providers observed that quality assurance visits to health facilities by vetting team to *“verify that expenditure claimed in the reimbursement forms corresponded with facility records”* (Policymaker 8, SHB) further adds to the delay. In addition, providers mentioned that *“scrutinizing volumes of claims submitted from all health districts by one central vetting committee took time”* (Provider 14, District B).

The delay in transferring funds to providers resulted from delay in approving vetted claims and transfer of funds from the PDPD to the State Health Board. Policymakers observed that the Chief Executive Officer of the Ministry of Health (commissioner) *“sat as an approving authority for the Steering Committee”* (Policymaker 1, PDPD), and reimbursement depended on *“whether the commissioner was willing to approve funds”* (Policymaker 6, SHB). Consequently, the reimbursement *“timelines stipulated in the free care programme guidelines were not met and reimbursements took more than six months after vetting”* (Policymaker 2, PDPD).

### Transparency

Most policymakers and providers indicated that the evidence of tax payment policy was poorly implemented in health facilities. Most providers stated that they resumed charging user fees since many users are unable to provide evidence of tax payment. District-level policymakers and providers interpreted evidence of tax payment to mean *“tax clearance”* (Policymaker 14, District B), *“tax receipts for three years”* (Provider 9, District B), payment of some money at the facility level in lieu of tax (Provider 1, District A) or presentation of *“pay slip or Gen 35”* (confirmation of public sector employment) (Provider 15, District B). Even though one HFC leaders observed that clients who are not civil servants are required to pay some money in lieu of tax, most HFC leaders claimed that the evidence of tax payment policy was not implemented in their health facilities because *“we (citizens) find it difficult to pay taxes”* (HFC leader 2, District A) and health workers do not create sufficient awareness about the taxation policy among users as *“providers capitalised on the taxation policy to close the chapter of free MCH services”* (HFC leader 6, District B).

Most policymakers spoke of the limited transparency in the business practices of the State Health Board in reimbursement of providers. For example, vetting officers were accused of inflating claims before recommending claims to the PDPD for approval. The PDPD constituted a Financial Monitoring Committee to provide oversight on disbursement of approved claims to health facilities and central medical store by the SHB. However, the State Health Board resisted financial oversight from the Financial Monitoring Committee. One policymaker revealed that, *“They instructed us that we (State Health Board) should never issue cheque to any facility without reporting to them, the so-called monitoring committee. We told them that we don’t know them. As soon as the money hits the account, we finish what we are supposed to do, we issue cheques to the facilities”* (Policymaker 8, SHB).

### Responsiveness

Participants of all categories claimed that the criteria and procedure for allocation of resources to health facilities are unclear. Most providers and HFC leaders claimed that the programme have collapsed since users still incurred out-of-pocket expenditure for basic maternal and child health services and drugs. *“It is already failing. No child between zero and five years is treated freely, no pregnant woman is treated freely, and no delivery is done freely in this facility”* (HFC leader 4, District A). It was explained that resource allocation to providers have not been based on needs. For example, providers *“have enough equipment…but the manpower and the space and infrastructure are lacking”* (Provider 14, District B) and *“when one visited a health facility drug store, one would see certain drugs that their half-life was about to expire, yet they were supplied”* (Policymaker 12, District B). Moreover, providers’ drug revolving fund (DRF) stocks were depleted due to delayed- or non-reimbursement of FMCHP drug expenditure.

### Equity and inclusiveness

All participants claim that more rural dwellers than urban residents were excluded from free maternal and child health services. Policymakers attributed this to the skewed distribution of health workers to urban districts, which resulted in shortages of health workers in rural health facilities. *“The distribution of health workers is more in favour of urban facilities, whereas rural facilities are starved of highly skilled staff”* (Policymaker 10, SHB). Providers stated that more health facilities in rural areas than urban areas have stopped providing free maternal and child health services following unpredictable provider payment. HFC leaders indicated that the policy of presenting evidence of tax payment resulted in lower use of free care services in rural districts than in urban districts.

### Effectiveness and efficiency

Some state-level policymakers stated that the Ministry of Health used FMCHP funds for purposes beyond the scope of the FMCHP. In the words of one policymaker “*if approval for maternal and child health-related program has been received from the Governor and is not cash-backed, we normally fund such programs from FMCHP pool*” (Policymaker 4, PDPD). In contrast, some district-level policymakers claim that FMCHP funds are misappropriated by public officials. A district-level policymaker stated that “*Some people decided to put their hands into that money and take away a chunk of it and that created a lot of problems*” (Policymaker 11, District B).

Some state-level policymakers and providers pointed out that LHA secretaries misused funds in LHA accounts meant for health facilities to replenish DRF items used for free care services during the early years of implementation. One provider observed that *“Cheques were coming straight to the LHA secretaries, and the cheques were supposed to be cashed and used to buy drugs, but somewhere along the line, some LHA secretaries tampered with the fund and the drug revolving fund stocks were de-capitalized”* (Provider 12, District B).

Many policymakers and providers further indicated that some service providers inflated claims in three ways – by billing beyond fee schedule, overreporting attendance and inclusion of services beyond scope of facilities or the FMCHP. A provider attested *“when they were paying the bill, one of our colleagues said, you people don’t know that you have to write names so that your money will come up”* (Provider 12, District B). HFC leaders were not aware of fund management in FMCHP but observed that monitoring of provider performance was irregular. One HFC leader said: *“you hardly see supervisors from the local government. The occasional supervisors we see are those of SURE-P and PATHS2 (development partners)”* (HFC leader 4, District A).

### Accountability

Participants of all categories stated that HFCs did not participate in formulating and implementing evidence of tax payment policy and provider payment system. A service provider stated that *“there was a circular issued by the Board directing that entitlement to free maternal and child health services be based on presentation of tax clearance”* (Provider 2, District A). On the other hand, they explained that HFCs and civil society organisations have argued for removal of evidence of tax payment. Participants of all categories also stated that most service providers lacked service charters and complaint box. Where service charter existed, most service providers were not creating sufficient awareness about service charters. In the words of one respondent, *“It is not just developing service chatter and pasting in the facility, it should be drummed into the ears of people”* (Policymaker 10, SHB).

### Information and intelligence

Most policymakers and providers explained that reimbursement processes are paper-based and neither driven by information and communication technology nor integrated into the state health management information system. Also, some policymakers, few providers and one HFC leaders explained that changes in the referral system and provider payment procedure were informed by analysis of data on reimbursement of providers and procurement at the central medical store. Initially, provider drug costs were paid through LHA secretaries, but currently *“providers collect drugs from the central medical store equivalent to approved facility expenditure on drugs”* (Policymaker 1, PDPD).

### Ethics

Policymakers and HFC leaders stated that providers rationed free care services and attended more promptly to clients that pay out-of-pocket than those seeking free care services. One policymaker noted that *“We have had to sanction one or two providers for collecting money from a patient instead of treating the patient under the free MCH programme”* (Policymaker 1, PDPD). Most providers explained that they resumed charging fees due to unpredictable reimbursement which resulted in the depletion of their drug revolving fund stocks needed to provide fee-exempt services. HFC leaders, nonetheless, argued that “*health workers do not support free services*” (HFC leader 12, District B) because the FMCHP reduced providers’ opportunity for informal payments. Few policymakers and providers claimed that service users referred to the State Teaching Hospital were denied access to free maternal and child health services *“using all manner of subterfuge to convert them into fee paying patients”* (Policymaker 6, SHB). Few providers also claimed that users requiring emergency care were exempt from evidence of tax payment before initiation of care. In contrast, HFC leaders argued that providers withheld care from needy clients because they are unable to present evidence of tax payment.

## Discussion

The study has examined how governance practices influence purchasing in the free maternal and child healthcare policy in Enugu state, Nigeria from the perspectives of policymakers, providers and citizens. The findings highlight, using Siddiqi et al. health systems governance framework, the key governance imperatives needed to scale-up strategic purchasing in free healthcare policies in resource-constrained settings.

This study revealed that institutional conflicts due to dysfunctional inter-organisational relationships among key actors resulted in absence of purchaser-provider split, which undermined the formal, accountable governance structure for purchasing. Contrary to policy, the MOH usurped the purchasing function of the Steering Committee and State Implementation Committee as with experiences in Seguro Popular [13, 16], but differs from evidence from Thailand, India, Vietnam and Nigeria [8–12]. Because of the dominant role of MOH, the capacity, power and responsibility of the Steering Committee and State Implementation Committee as strategic purchasers diminished. Improving strategic vision in free healthcare schemes would entail establishing an autonomous statutory body, separate from the Ministry of Health, with sufficient financial leverage over providers and empowered to enforce accountability by providers through active purchasing.

The findings of this study that no clear framework for accrediting, contracting and monitoring providers exists is similar to absence of accreditation of health facilities in the federal free healthcare policy in Nigeria and limited mandatory accreditation of providers in Mexico’s Seguro Popular but contrasts the regular hospital accreditation and formal contracting of providers in Thailand’s universal coverage scheme (UCS) and China’s NCMS [8, 9, 12, 14, 15]. The purchasing agency, as a strategic purchaser, should select primary care providers and referral hospitals, which could be public or private, with explicit performance contract between the purchaser and providers indicating the selection criteria, service entitlements, provider payment system and routine performance monitoring system [4]. Besides, registering beneficiaries with accredited primary care providers would provide basis for capitating providers [15].

This study highlighted two constraints to participation and consensus orientation. First, changes in coverage conditions in the FMCHP were not based on wider stakeholder involvement. The decision to use evidence of tax payment as rationing strategy excluded providers and citizens, which differs from participatory-evidence-based-contestable benefit package design process adopted in Thailand’s UCS [31]. Secondly, Local Health Authority (LHA) Secretaries were excluded from routine provider performance monitoring. Dysfunctional inter-organisational relationship between the Ministry of Health and Local Health Authorities resulted in withholding of LHAs’ administrative costs that enables LHAs monitor FMCHP implementation at facility level. An improved governance model would require inclusive dialogue and involvement of stakeholders in designing benefits, payment of providers and provider performance monitoring.

Consistent enforcement of provider payment standards is a key governance requirement for moving towards strategic purchasing which deserves attention as the findings of this study highlighted three types of delay in payment of providers. When providers are not paid at defined times, they lose motivation, and employ discretion to modify policy through implementation. Equally, funds accumulate, and the accumulated funds may discourage the government from timely transfer of contributions to the FMCHP pool. The delay in transferring funds from state to healthcare providers has also been seen in Mexico due to political factors, such as negotiations of resource allocations [13]. Since the typology of delays in provider payment in the FMCHP is varied, it might be helpful for the purchasing agency to improve the capacity of providers to prepare claims, strengthen the vetting team, enforce the purchasing rules and monitor payment of providers.

This study’s findings underscored importance of transparency in entitlements to free services and obligations to users. Many providers do not seem to create sufficient awareness among users about the evidence of tax payment. Yet, other providers required users to pay some money in lieu of tax at the point of service delivery. Sadly, the evidence of tax payment policy resulted in effective return of user fees in many health facilities, increasing the likelihood that the poor may not be protected from financial catastrophe. Residence-based entitlement could be adopted, in which pregnant women and under-5 children enrol with the network of primary health care providers within their localities as with experiences in Thailand, Mexico and China [8, 9, 14]. To register with providers, eligible beneficiaries may require proofs of residency such as certification letter by health facilities committee members, town union leaders or traditional rulers; or public utility bills consistent with experiences in Thailand’s UCS [8, 31].

This study further revealed that the criteria and procedure for resource allocation to health facilities are not clear, resulting in low responsiveness of health facilities to free healthcare users. The finding that resource allocation to health facilities providing free services are not based on needs is similar to findings from Ghana, Kenya and Nigeria [12, 32, 33] but contrasts experiences in Thailand [31]. Beside service delivery infrastructure gaps in provider facilities, delay in payment of providers resulted in untimely replenishment of facility drug stocks, necessitating health workers’ poor adherence to free care policy and increased involvement in parallel drug supply system. Scaling-up strategic purchasing must incorporate strengthening service provision and quality.

This study findings that the FMCHP excluded some target beneficiaries is consistent with evidence from Senegal [34]. Three key factors drive inequity and social exclusion in the FMCHP. First, skewed distribution of health workers to urban districts resulted in shortages of health workers in rural health facilities. Secondly, the FMCHP seem to have collapsed in some health facilities due to unpredictable provider payment. Thirdly, as the poor do not seem to pay taxes, evidence of tax payment resulted in low use of free care services in some health facilities especially in rural areas. Improving staffing of health facilities, timely payment of providers and delink entitlements to free healthcare from taxation policy would address the equity goal of the free care policy.

This study further confirmed the contradictory incentive environment created by fee-for-service payment method for purchasers and providers to manage their expenditure, make efficient use of resources and improve quality. The Ministry of Health used the FMCHP funds for unauthorised purposes similar to experiences in Mexico’s Seguro Popular [13, 16]. Providers also seemed to inflate FMCHP claims by over-billing free services. Moreover, there are allegations of gaming with FMCHP claims by the district officials and vetting team. It might be more efficient and fiscally sustainable to provide capitated funds for every pregnant woman and under-5 children that registers with primary care providers rather than refunding costs on itemised fee-for-service as is the case in Thailand and Vietnam [8, 11]. Referral services can be paid for using diagnostic-related groups (DRG) with provider payment cap consistent with transition from fee-for-service to DRG in Ghana and Thailand’s UCS [8, 18]. Adopting a mixed provider payment system and adherence to the fund management rules might improve the effectiveness and efficiency of strategic purchasing.

Citizen participation in designing benefits, reporting provider claims and monitoring provider payment was weak in this study. Weak social accountability initiatives were also observed in India’s RSBY, Mexico’s Seguro Popular and Nigeria’s NHIS-MDG free healthcare policy [12, 35–37], which contrasts active citizen participation in Thailand’s UCS [8, 31]. Effectiveness of social accountability in purchasing was limited by weak legal framework, moribund FMCHP committees, restricted financial information disclosure, and distrustful relationship with policymakers and providers as we reported elsewhere [1]. Scaling up strategic purchasing in FMCHP would require citizens to play stronger roles in defining benefits and provider payment process.

The study findings highlight the crucial role of information management system for effective purchasing. Two institutional designs of FMCHP changed during implementation based on information and intelligence. First, the referral system was strengthened by designating the state teaching hospital a referral facility. Secondly, the change in provider payment process from use of Local Health Authorities as financial intermediaries to paying health facilities directly. Nevertheless, a persisting governance challenge is to transit from paper-based system to a system driven by information and communication technology (ICT). The use ICT infrastructure, aligned with the health management information system, to manage provider payment process would ensure transparent and efficient management of FMCHP claims and provide data on routine attendance that could be used to design DRG payment system [38].

The study findings emphasise the need to enforce high ethical standards in facilities providing free healthcare services. Two factors that undermined ethics in this study include providers’ under-provision of free health services and users’ non-adherence to primary care gatekeeping role. Preference for ‘fee-paying’ patients and less attention to fee-exempt users indicate undesired reactions of providers to uncertain provider payment and conflictive policy linking evidence of tax payment to benefits but may also be a driving factor for non-adherence to referral rules. The finding of low adherence to referral guidelines is consistent findings in Thailand and Vietnam, where users, who by-pass referral system, pay out-of-pocket for free health services [8, 11]. Promoting ethical standards would require attention to the provider payment methods, delinking of user obligations from benefits and enforcement of gatekeeping rules.

This study adds to policy debate on UHC by providing useful insights into the institutional designs and organisational practices that shape purchasing in user fee removal policies in resource-limited settings. As this study was limited to one sub-national context, the views expressed by participants may not be generalizable to other stakeholders. However, as the purpose was not generalization but to understand how governance practices shape purchasing system in free healthcare policies in low resource settings, the study used rich descriptions grounded in participants’ experiences and a variety of scientific practices to ensure trustworthiness of findings [39, 40].

## Conclusions

This study emphasizes a need for appropriate governance model for strategic purchasing in free healthcare policies in Nigeria and similar settings. Even though the free healthcare policies have huge potential to contribute towards universal health coverage, weak governance of purchasing function is a key limiting factor. The governance model should incorporate clear mandate and objectives for strategic purchasing; sufficient purchaser autonomy; clear framework for selective contracting; inclusive dialogue and stakeholder involvement; transparent benefit package, delinked from user obligations; need based resource allocation to health facilities; adoption of ICT-driven, coherent mixed provider payment systems; enforcement of provider payment rules; stronger roles for citizens; enforcement of gatekeeping rules; and use of data for wider system monitoring and decision-making.

## Additional file

Additional file 1: Assessment of governance of the free maternal and child healthcare programme in Enugu State, Nigeria: interview guide for key actors. The tool was used to guide data collection from key actors involved in the implementation of the free care policy at the state and district levels including revenue generation, pooling and fund management, purchasing and provision of free services. However, the data reported in this paper included only data related to purchasing function. (DOCX 15 kb)

## Abbreviations

DRF: Drug revolving fund
DRG: Diagnostic related groups
ETP: Evidence of tax payment
FMCHP: Free maternal and child healthcare programme
HFC: Health facility committees
HOD: Head of department
HTA: Health technology assessment
LHA: Local Health Authority
MOH: Ministry of health
PHC: Primary health care
REPSS: State Health Protection Regime
SHB: State Health Board
UCS: Universal coverage schemes
UHC: Universal health coverage
