# Supplementary material for: Scaling-up strategic purchasing: analysis of health system governance imperatives for strategic purchasing in a free maternal and child healthcare programme in Enugu State, Nigeria
Source: BMC Health Serv Res. 2018 Apr 5;18:245. doi: 10.1186/s12913-018-3078-x (PMC5887245; doi:10.1186/s12913-018-3078-x)
Supplement: Supplementary file 1 — Assessment of governance of the free maternal and child healthcare programme in Enugu State, Nigeria: interview guide for key actors. The tool was used to guide data collection from key actors involved in the implementation of the free care policy at the state and district levels including revenue generation, pooling and fund management, purchasing and provision of free services. However, the data reported in this paper included only data related to purchasing function. (DOCX 15 kb) [file 12913_2018_3078_MOESM1_ESM.docx]

**ASSESSMENT OF GOVERNANCE OF FREE MATERNAL AND CHILD HEALTHCARE PROGRAMME (FMCHP) IN ENUGU STATE, NIGERIA: INTERVIEW GUIDE FOR KEY ACTORS**

| **Questions** | **Probes** |
| --- | --- |
| Could you please introduce yourself and briefly describe your work | Probe for role in FMCHP |
| 1. What contextual factors necessitated introduction of FMCHP? | Ask for social, economic, political, environmental or health systems factors |
| 1. What are the main objectives and characteristics of FMCHP? 2. How have the objectives and characteristics changed overtime? | Ask for policy changes in revenue generation, pooling, service entitlement, obligations of consumers |
| 1. How were design features or changes communicated to all actors? 2. How has policy communication influenced implementation of FMCHP? | Ask for use of hard copies, meetings, training, orientation, media |
| 1. Who are the actors that play key roles in making decisions about FMCHP? 2. What role does each play or not play in FMCHP and why? 3. Is there a clear, well defined policy and long-term vision for improving FMCHP? 4. Are different stakeholders’ views considered in decision-making regarding FMCHP? | Ask for roles in revenue generation, pooling, purchasing and providing resources to health facilities.  Ask for legal framework |
| 1. How does each FMCHP committee execute its roles? | Probe for discretion, authority, tools, decision space and resources to execute roles; |
| 1. What was the plan for funding FMCHP and how has this plan been implemented? | Ask for sources and level of funding; how funds flow from contributors to Ministry of Health; |
| 1. How does government ensure that FMCHP funds are used for what they are meant? 2. Are data on finance and administration of FMCHP readily available and is the process transparent? 3. Any stipulations about administrative costs and how well have these been implemented? |  |
| 1. What mechanism(s) exist to hold providers accountable to MoH in FMCHP implementation? 2. Are monitoring and supervision of FMCHP conducted as per guidelines? 3. Are financial regulations/ reimbursement standards enforced as defined in implementation guidelines? 4. How does information flow from MOH to providers and vice versa and what enables or constrains information flow and use? | How funds flow from MOH to providers (provider reimbursement) and role of districts/Local health authorities. |
| 1. What enables or constrains resource availability for FMCHP at health facilities? 2. Is needs assessment part of the resource allocation process and how are findings used? | Probe for impact of FMCHP on functioning of health facilities. |
| 1. Are there disparities in access to free maternal and child health services among poor and non-poor, urban and rural households? 2. How are standards of maternal and child health services promoted and enforced? |  |
| 1. What mechanisms exist to hold the MOH directly accountable to citizens for optimal implementation of FMCHP? 2. What enables or constrains citizens’ participation in FMCHP implementation? 3. What type of consumer complaint mechanisms exist and to what extent are they being used in FMCHP/ provider facilities? | Probe for involvement of CSOs/health facility committees in FMCHP and use of service charter.  Probe for awareness of benefits and obligation. |
| 1. What is your general impression about FMCHP 2. In your views, what will be the most meaningful changes to improve implementation of FMCHP? |  |
| Thank you for your participation | |
